# Fahr syndrome associated with hypoparathyroidism: Case report

**DOI:** 10.1097/MD.0000000000046742

**Published:** 2025-12-19

**Authors:** Bo Zhao, Jiawei Li, Jinyan Yu, Ying Wang, Xueming Pang

**Affiliations:** a Department of Neurology, Hangzhou Linping District Hospital of Integrated Traditional Chinese and Western Medicine, Hangzhou, China.

**Keywords:** epilepsy, Fahr syndrome, hypoparathyroidism

## Abstract

**Rationale:**

Fahr’s syndrome is characterized by bilateral intracranial calcifications involving the cerebellum, basal ganglia, thalamus, radial crown, and subcortical white matter. It is a rare neurological disorder often associated with hypoparathyroidism (HypoPT). HypoPT-induced hypocalcemia triggers calcium-phosphate deposition in brain parenchyma, typically manifesting as tetany, seizures, or extrapyramidal symptoms. Diagnostic challenges arise from overlapping symptom features with neurodegenerative and psychiatric disorders. We present a case of Fahr’s syndrome secondary to iatrogenic HypoPT, highlighting pitfalls in long-term misdiagnosis.

**Patient concerns:**

A 58-year-old female presented with progressive rigidity, resting tremor, and recurrent tetanic spasms for 2 years. She had undergone neck surgery 18 years ago and had a 15-year diagnosis of epilepsy.

**Diagnoses:**

During hospitalization, her laboratory results indicated significant hypocalcemia, accompanied by extensive intracranial calcification on head computed tomography. Combining her symptoms, signs, and test results, we finally diagnosed her with Fahr’s syndrome associated with HypoPT.

**Interventions:**

The patient received intravenous calcium gluconate (1 g/day) and oral 0.5 μg calcitriol with 600 mg calcium carbonate twice daily.

**Outcomes:**

Serum calcium normalized within two weeks, with resolution of tetany and creatine kinase reduction to 210 U/L. In addition, the patient’s symptoms, such as rigidity and tremor of extremities, along with bradykinesia, also got relieved by the correction of calcium.

**Lessons:**

For neurologists, when encountering patients presenting with clinical seizures or movement disorders, prompt head computed tomography scans and assessments of serum calcium, phosphorus, and parathyroid hormone levels are crucial to facilitate early diagnosis.

## 1. Introduction

Fahr syndrome is a sporadic or familial disorder reported by Fahr in 1930. Fahr disease, also known as familial idiopathic basal ganglia calcification, is often manifested as a family susceptibility, autosomal dominant inheritance, typically manifesting in adolescents or adults.^[1]^ Fahr syndrome arises from intracranial calcifications secondary to etiologies including primary or secondary HypoPT, pseudohypoparathyroidism, infectious diseases, neurodegenerative disorders, mitochondrial disorders, and systemic conditions, with HypoPT being the most frequent cause.^[2]^

HypoPT, a rare metabolic disorder characterized by hypocalcemia and low or undetectable levels of parathyroid hormone (PTH),^[3]^ often presents with neuropsychiatric symptoms, leading to frequent misdiagnosis, particularly among neurologists.^[4]^ Systemic calcifications may affect multiple organs, most commonly the kidneys, but also joints, eyes, skin, and the vascular system. Intracranial calcifications, though rare, have been reported.^[5]^ To date, the most prevalent etiology is surgical removal of the anterior neck or parathyroid damage,^[6]^ while autoimmune destruction and genetic defects account for most remaining cases.^[7]^ Herein, we present a case of Fahr syndrome secondary to HypoPT.

## 2. Case presentation

### 2.1. Patient information and clinical findings

A 58-year-old female was brought by her daughter in September 2024. Her complaints included severe tetany involving the facial muscles and body muscles, rigidity and tremor of extremities and trunk over the past 2 years. She had undergone neck surgery 18 years ago and carried a 15-year diagnosis of epilepsy managed with valproate (500 mg/day) and carbamazepine (200 mg/day). Due to the long duration of the surgery, the patient was unable to provide the specific surgical process and records. Neurological examination revealed bradykinesia, spastic quadriparesis, and positive Chvostek/Trousseau signs. She had no notable family history, thyroid disease, hypertension, diabetes, stroke, central nervous system infections, or autoimmune diseases. In order to determine whether it was Parkinson disease, she was admitted to our hospital.

### 2.2. Laboratory test and imaging results

The results of the major laboratory tests are shown in Table 1. The most striking feature was severe hypocalcemia (1.17 mmol/L, normal range 2.20–2.65 mmol/L), hyperphosphatemia (2.64 mmol/L, normal range 0.81–1.45mmol/L), and mild hypomagnesemia (0.60 mmol/L, normal range 0.77–1.03mmol/L), meanwhile, intact PTH was decrease to < 6.30 ng/L (normal range 18.5–88.0 ng/L), and 25 (OH) vitamin D was normal. However, it was important to note that Creatine Kinase (CK) raised to 3933 U/L (normal range 0–164 U/L), which indicated a serious muscle damage. In addition, the patient tested normal for adrenocorticotropic hormone(ACTH), sex hormone (including estradiol, follicle stimulating hormone, prolactin, testosterone, luteinizing hormone and progesterone), and serum tumor markers.

**Table 1:** The major laboratory test results.

Test name	Result	Unit	Reference range
Calcium	1.17	mmol/L	2.20–2.65
Phosphorus	2.64	mmol/L	0.81–1.45
Sodium	140	mmol/L	137–147
Chloride	101	mmol/L	99–110
Potassium	3.58	mmol/L	3.50–5.30
Magnesium	0.60	mmol/L	0.77–1.03
Intact PTH	<6.30	ng/L	18.5–88.0
25 (OH) vitamin D	29.34	ng/mL	≥20.00
ALT	16	U/L	0–35
TSH	1.37	uIU/mL	0.56–5.91
FT3	3.26	pg/mL	2.50–3.90
FT4	0.99	pg/mL	0.61–1.12
TLC	8.5	109/L	3.5–9.5
Platelet count	205	109/L	125–350
Uric acid	205	μmol/L	Male: 208–428, Female: 154–357
HCY	10.9	μmol/L	0.0–15.0
Creatinine	69	μmol/L	49–90
CK	3933	U/L	0–164
ACTH	30.52	pg/mL	7.00–65.00

ACTH = adrenocorticotropic hormone, ALT = alanine aminotransaminase, HCY = homocysteine, CK = creatine kinase, PTH = parathyroid hormone, TSH = thyroid stimulating hormone, TLC = total leukocyte count.

**Figure 1.**
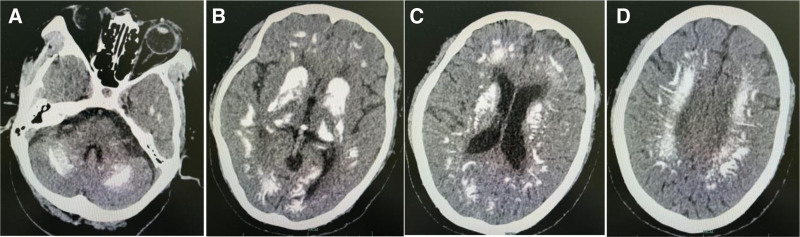
Brain CT showed bilateral symmetrical calcification: (A) cerebellum; (B) corpus striatum, thalami and occipital lobe; (C) frontal lobe and paraventricular white matter; (D) centrum semiovale of both cerebral hemispheres.

**Figure 2.**
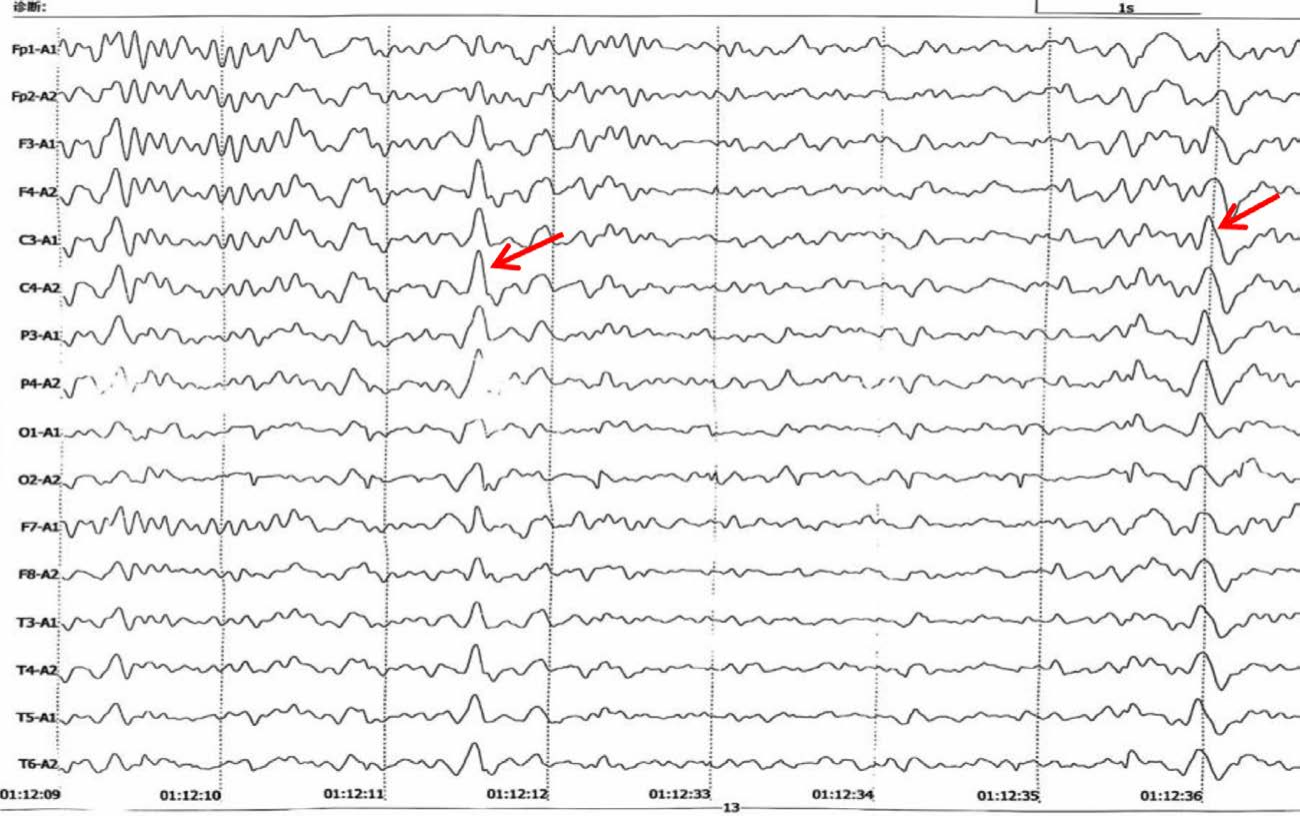
Sharp wave attacks can be seen in all leads (as indicated by the red arrow), the bilateral cerebral hemispheres are symmetrical.

Cranial CT showed widespread bilateral symmetrical regions of calcification in the cerebellum, basal ganglia, and subcortical white matter (Fig. 1). Thyroid sonography did not reveal any abnormal findings, and parathyroid glands sonography showed no enlarged parathyroid gland echo. Results from 32 channel digital electroencephalography (EEG) was performed, and revealed interictal sharp waves (Fig. 2).

### 2.3. Treatment and follow-up

The patient received intravenous calcium gluconate (1 g/day) and oral 0.5μg calcitriol with 600mg calcium carbonate twice daily. Furthermore, because she had not experienced seizures in years, she received a low dose of valproate sodium 500 mg/day. Serum calcium normalized within 2 weeks, with resolution of tetany and CK reduction to 210 U/L. In addition, the patient’s symptoms, such as rigidity and tremor of extremities, along with bradykinesia, also got relived by correction of calcium.

## 3. Discussion

Epilepsy, one of the most common brain conditions, affects over 70 million people worldwide.^[8]^ Too often, people are categorized as simply having epilepsy whereas the causes of epilepsy are complex: genetic, structural, metabolic, infectious, immune, and unknown.^[9]^ Currently, the treatment for epilepsy patients, on the one hand, focuses on etiological treatment, and on the other hand, antiepileptic drug is necessary. For this patient, her EEG revealed interictal sharp waves, which can explain the symptomatic epilepsy, but the etiology is metabolic encephalopathy rather than true epilepsy. There were no more epileptic seizures after actively supplementing calcium and administering antiepileptic drugs during the hospitalization.

The parathyroid glands are one of the endocrine glands which are in close proximity to the thyroid gland and often share a common blood supply, and their main function is to secrete PTH. It causes HypoPT when its function is impaired. The most common cause of HypoPT is complication of surgery, others including autoimmune disease, genetic causes, infiltrative diseases, mineral deposition or due to abnormalities in serum levels of magnesium.^[10]^ The small size, color and shape of a parathyroid gland can make it difficult to distinguish from surrounding tissues such as fat and cervical lymph nodes, so it is not uncommon for the parathyroid gland to be injured or accidentally removed during anterior neck surgery, which can cause transient or permanent HypoPT.^[11]^ In this case, the patient had undergone neck surgery 18 years ago, and due to the long duration of the surgery, the patient was unable to provide the specific surgical process and records. However, we suspect that this might have damaged the patient’s parathyroid glands, for the parathyroid glands sonography did not reveal any swollen or infiltrative diseases. In addition, considering that the patient is 58-year-old female and has recurrent epilepsy, which is a high-risk age for autoimmune diseases, it is speculated that autoimmune factors may not be ruled out either. Unfortunately, no relevant autoimmune tests were conducted during the patient’s hospitalization.

## 4. Conclusion

This case illustrates 3 critical teaching points. First is the etiological link. Postsurgical HypoPT following neck procedures is a well-documented cause of Fahr syndrome.^[12]^ The 18-year latency between neck surgery and symptom onset aligns with chronic hypocalcemia’s role in calcification pathogenesis.^[13]^ The second is the diagnostic pitfall. Misdiagnosis as epilepsy persisted for 15 years, emphasizing the need to reassess seizure etiology in HypoPT patients. Interictal EEG abnormalities may reflect metabolic encephalopathy rather than true epilepsy. The last one is biochemical-clinical correlation. Markedly elevated CK (3933 U/L) indicated hypocalcemia-induced rhabdomyolysis, a rare but life-threatening complication.^[14]^ Fahr syndrome should be considered in patients with chronic hypocalcemia and unexplained neuropsychiatric symptoms. Early measurement of calcium-phosphorus balance and PTH levels and necessary cranial CT scan are critical to avoid diagnostic delays.

There are some limitations of our study. We had no way to determin the type of the patient’s previous neck surgery and the specific surgical method. Moreover, due to the small volume of the parathyroid gland and its difficulty in distinguishing it from the surrounding tissues, in this patient, the color Doppler ultrasound only described that no enlarged parathyroid gland echo was observed, but it was impossible to determine whether the parathyroid glands were damaged. Therefore, the cause of HypoPT in this patient was actually impossible to determine.

For future works, we should focus on the diagnosis and treatment of HypoPT. In terms of diagnosis, a screening and diagnostic process for neurological complications related to HypoPT should be established, to help neurologists identify HypoPT quickly when facing unexplained epilepsy, brain calcification, and movement disorders. In terms of treatment, which is better, traditional calcium/vitamin D therapy or PTH replacement therapy? Is the brain calcification reversible? These issues are all worthy of exploration by clinicians. In addition, a multidisciplinary collaboration (MDT) diagnosis and treatment model involving neurology, endocrinology, psychiatry and rehabilitation should be established, and its effect on improving the overall prognosis of patients should be evaluated.

## Author contributions

**Data curation:** Ying Wang.

**Investigation:** Jinyan Yu.

**Supervision:** Jiawei Li.

**Writing – original draft:** Bo Zhao.

**Writing – review & editing:** Xueming Pang.
